# Chinese families with autosomal recessive hereditary spastic paraplegia caused by mutations in SPG11

**DOI:** 10.1186/s12883-019-1593-y

**Published:** 2020-01-03

**Authors:** Xueping Chen, Jiao Liu, Qian-Qian Wei, Ru Wei Ou, Bei Cao, Xiaoqin Yuan, Yanbing Hou, Lingyu Zhang, Huifang Shang

**Affiliations:** Department of Neurology, West China Hospital, Sichuan University, No. 37 Guoxue Xiang, Chengdu, 610041 Sichuan China

**Keywords:** Autosomal recessive hereditary spastic paraplegia, Spastic paraplegia type 11, Genetic spectrum, Phenotypic spectrum, Autosomal-recessive juvenile amyotrophic lateral sclerosis

## Abstract

**Background:**

Spastic paraplegia type 11 (*SPG11*) mutations are the most frequent cause of autosomal recessive hereditary spastic paraplegia (ARHSP). We are aiming to identify the causative mutations in *SPG11* among families referred to our center with ARHSP in a Chinese population.

**Methods:**

Targeted next-generation sequencing was performed on the patients to identify disease-causing mutations. Variants were analyzed according to their predicted pathogenicity and their relevance to the clinical phenotypes. The segregation in the family members was validated by Sanger sequencing.

**Results:**

A total of 12 mutations in *SPG11* gene from 9 index cases were identified, including 6 frameshift mutations, 3 missense mutations, 1 nonsense mutation, 1 splicing mutation, and 1 intron deletion mutation. In 6 of these patients, the mutations were homozygous, and the other 3 patients carried two compound heterozygous mutations. Six mutations were novel; 2 were classified as pathogenic, 1 were considered as likely pathogenic, and the other 3 were variants of unknown significance. Additionally, 1 missense heterozygous variant we found was also carried by amyotrophic lateral sclerosis (ALS) patient. Clinically and electrophysiologically, some of our ARHSP patients partially shared various features of autosomal-recessive juvenile amyotrophic lateral sclerosis (ARJALS), including combination of both UMN and LMN degeneration.

**Conclusions:**

The results contribute to extending of the *SPG11* gene mutation spectrum and emphasizing a putative link between ARHSP and ARJALS.

## Background

Hereditary spastic paraplegia (HSP), characterized by progressive weakness the lower limbs and spastic paraplegia, is a rare group of neurodegenerative diseases with high clinical and genetic heterogeneity [1]. Clinically, HSP can be divided into “pure” forms if spastic paraplegia is the major presenting feature and “complicated” forms if additional neurological or systemic abnormalities are exhibited, including ataxia, cognitive decline, epilepsy, peripheral neuropathy, and so on [2]. Genetically, based upon mode of inheritance, HSP can be classified as autosomal dominant, autosomal recessive, X-linked recessive, and sporadic. Spastic paraplegia type 11 (*SPG11*) mutations are the most frequent form of autosomal recessive hereditary spastic paraplegia (ARHSP). Pathologically, the mutations of *SPG11* primarily cause progressive degeneration of the upper motor neurons (UMNs), but lower-limb muscle atrophy and fasciculations are also observed in patients carrying *SPG11* mutation, indicating the involvement of lower motor neuron (LMN). Furthermore, LMN alterations can be found on electromyography (EMG) [3–5]. A characteristic manifestation of SPG11 is the presence of a thin corpus callosum (TCC) in the MRI scanning [6], and *SPG11* gene mutations account for 41 to 77% of ARHSP-TCC cases [1, 3, 7, 8]. The involvement of *SPG11* in UMNs and LMNs degeneration is further underlined by the finding that pathogenic mutations of *SPG11* have also been identified in patients with autosomal-recessive juvenile amyotrophic lateral sclerosis (ARJALS) [9].

*SPG11* maps to chromosome 15q21, and has 100,982 coding nucleotides which are translated into a protein with 2443 amino acid, SPATACSIN. This protein is particularly expressed in the neurons of the cerebellum and cerebral cortex [7]. More 100 pathogenic mutations have been reported, such as frameshift, missense, large-scale deletion, splice-site mutations, and small truncating indels [3, 8, 10, 11]. The mutations can lead to absence or functional impairment of SPATACSIN protein. Screening of *SPG11* mutations is essential to obtain the genotype-phenotype correlations in SPG11.

The development of targeted next-generation sequencing (NGS) is enabling us to investigate the large targeted proportions of the genome in a rapid, affordable, and comprehensive way. In the present study, we applied targeted NGS combined with multiplex ligation-dependent probe amplification (MLPA) for the genetic analysis of a cohort of Chinese patients with suspected diagnosis of HSP. Patients carrying *SPG11* mutations were clinically and genetically examined, trying to ascertain the spectrum of mutations in *SPG11*, enlarge the ethnic origin of SPG11 patients, and provide additional clinical phenotypes resulting from *SPG11* mutations and more information on this genetically heterogeneous disease.

## Materials and methods

### Subjects

Detailed clinical assessments were performed and familial history and neurological features at onset were ascertained by interviews with the patients and the relatives, especially the HSP core symptoms. Patients were diagnosed with HSP on the basis of Harding’s criteria [12], and patients whose family histories were compatible with an autosomal recessive inheritance were included. A total of 32 ARHSP patients were recruited from the Department of Neurology, West China Hospital of Sichuan University, between January 2013 and December 2018. Ancillary investigations, including biochemical tests, electrophysiologic tests, and MRI scans were also conducted. In addition, 500 healthy subjects of matched geographical ancestry were recruited as controls. Written informed consent was obtained from all subjects engaged in this study and this study was approved by Ethic Committee of West China Hospital, Sichuan University.

### Targeted next-generation sequencing and MLPA analysis

Genomic DNA was extracted from peripheral leucocytes using standard procedures. Targeted next-generation sequencing was performed on genomic DNA samples of all the patients. The clinical exome panel included 85 targeted genes related to HSP and other inherited peripheral neuropathies (Kingmed Center for Clinical Laboratory CO., LTD, Guangzhou, China). The data were analyzed and the variants were filtered using the GATK best practises pipeline. The variants were annotated using the ANNOVAR (http://wannovar.usc.edu/) software. Variants with overall population allele frequencies of > 1% (0.01) in any of the public databases (1000 genome dataset, 6500 exome variant server, and Exome Aggregation Consortium database) were removed. In silico tools PolyPhen-2 (http://genetics.bwh.harvard.edu/pph2/index.shtml), SIFT (http://sift.jcvi.org/www/SIFT_seq_submit2.html), and Mutation Taster (http://www.mutationtaster.org/) were used to assess the pathogenicity of the variants. Initially, targeted exome sequencing was performed on the proband. A mean exome coverage of more than 98.1% was obtained, with a variant accuracy of more than 95%. PCR amplification using Sanger sequencing was further performed to verify the variants identified in the proband, and co-segregation analysis was performed among family members. Then, the candidate variants were classified according to the American College of Medical Genetics and Genomics (ACMG) Standards and Guidelines [13]. The MLPA analyses were performed in our ARHSP cohort comprised 32 probands for detection of large deletions or copy number variations, and *SPG11* deletions and duplication had not been identified our patients.

## Results

### Genetic findings

A total of 9 index cases with *SPG11* gene mutations were identified out of unrelated HSP patients, and 12 mutations were identified, including 6 frameshift mutations, 3 missense mutations, 1 nonsense mutation, 1 splicing mutation, and 1 intron deletion mutation. Of these, 6 variants were previously described and 6 variants were novel. According to the ACMG guidelines, 1 novel variant were classified as pathogenic, 1 novel variants were considered as likely pathogenic, 2 novel variants were considered as pathogenic, and the other 3 were variants of unknown significance. In 6 of these patients, the mutations were homozygous, and the other 3 patients had two compound heterozygous mutations (Table 1).

**Table 1:** Molecular analysis of gene mutations in *SPG11*

Patient	Type	Location	Variant	SIFT/PolyPhen/MutTaster	ExAC Allele Frequency	Clinical Features	Predicted Amino Acid Change	Mutation Type	Pathogenicity class according to ACMG	Reference
Family 1	compound heterozygous	Exon 33	c.6284 T > C^a^	Deleterious/deleterious/deleterious	0.0097	Spastic paraparesis, dysarthria, amyotrophy of the distal right hand muscles	p.(Leu2095Ser)	Missense	PM1 + PM2 + PM3 + PP3 + PP4 (likely pathogenic)	Kim, et al.
		Exon 6	c.1203 delA	Unknown/unknown/ unknown	0.00009		p.(Lys401fs)	Frameshift	PVS1 + PS1 + PM2 + PM3 + PP4 (pathogenic)	Denora, et al.
Family 2	compound heterozygous	Intron24	c.4162-10 T > G	Unknown/unknown/ unknown	0	Spastic cerebellar ataxic gait, intentional tremor of left hand, cognitive impairment	–	Missense	PM1 + PM2 + PM3 + PP4 (likely pathogenic)	Novel
		Exon11	c.2163dupT	Unknown/unknown/ unknown	0		p. (Ile722fs)	Frameshift	PVS1 + PM2 + PM3 + PP4 (pathogenic)	Liao, et al.
Family 3	homozygous	Exon 4	c.733_734del		0	Walking difficulties, spastic paraparesis, dysarthria, cognitive impairment	p. (Met245fs)	Frameshift	PVS1 + PS1 + PM2 + PP4 (pathogenic)	Stevanin, et al.
Family 4	homozygous	Exon 22	c.3805dupA	Unknown/unknown/ unknown	0	Spastic paraparesis, limb pain, cognitive impairment	p.(Arg1269fs)	Frameshift	PVS1 + PM1 + PM2 + PP4 (pathogenic)	Novel
Family 5	compound heterozygous	Exon2	c.317C > A	Deleterious/deleterious/benign	0	Walking difficulties, spastic paraparesis, tremor in the upper limbs, cognitive impairment	p. (Ala106Asp)	Missense	PM2 + PP1 + PP3 + PP4 (VUS)	Novel
		Intron20	c.3520 + 3_3520 + 6del	Unknown/unknown/ unknown	0		–	Intron deletion	PM2 + PP1 + PP4 (VUS)	Novel
Family 6	homozygous	Exon 38	c.6856C > T	Unknown/unknown/ deleterious	0.0002	Gait abnormality, spastic paraparesis, dysarthria, cognitive impairment	p.(Arg2286X)	Nonsense	PVS1 + PS1 + PM2 + PP4 (pathogenic)	Denora. et al.
Family 7	homozygous	Intron 39	c.7151 + 4_7151 + 7delAGTA	Unknown/unknown/ unknown	0	Gait abnormality, spastic paraparesis, cognitive impairment	–	splicing	PS1 + PS3 + PM2 + PP4 (pathogenic)	Liao, et al.
Family 8	homozygous	Intron 21	c.3686 + 3_3686 + 6delGAGT	Unknown/unknown/ unknown	0	Spastic paraparesis, cognitive impairment, walking difficulties	–	Frameshift	PM2 + PP4 (VUS)	Novel
Family 9	homozygous	Exon 7	c.1561_1562delAA	Unknown/unknown/ unknown	0	Spastic cerebellar ataxic gait, cognitive impairment, urinary disturbances, dysarthria, dysphagia, distal amyotrophy of lower limbs	p.(Asn521Trpfs)	Frameshift	PVS1 + PM1 + PM2 + PP4 (pathogenic)	Novel

*VUS* Variants of unknown significance, *PVS* Very strong evidence of pathogenicity, *PS* Strong evidence of pathogenicity, *PM* Moderate evidence of pathogenicity, *PP* Supporting evidence of pathogenicity; ^a^also has been identified in a ALS patients

### The genotypes and the clinical features of SPG11 patients

#### Family 1

Family 1: c.6284 T > C/p.(Leu2095Ser) and c.1203delA/p.(Lys401fs). In the index case, the variants were in a compound heterozygous configuration. The unaffected father was shown to be heterozygous for variation c.6284 T > C, and the unaffected mother was shown to be heterozygous for variation c.1203delA. The missense mutation c.6284 T > C located in exon 33 is predicted to lead to an amino acid change at position 2095 from Leu to Ser. A previous study enrolled 148 patients with a clinical diagnosis of sporadic ALS, found one ALS patient carried the variation c.6284 T > C [14]. The c.1203delA variant located in exon 6 leads to a frameshift mutation, which could introduce a premature termination codon and was predicted to cause the absence of the SPASTACSIN protein. The same c.1203delA mutation in heterozygous form was reported in previous studies [8, 10]. The index case is a 23-year-old woman, and she presented with a 9-year history of a progressive stiffness of the right upper extremity and difficulties in writing. Seven year ago, she developed progressive weakness of the lower extremities, gait disturbance, and slurred speech. The symptoms were becoming more and more severe, and by the time of hospitalization, the patient showed symptoms of walking difficulties with scissors gait. Neurological examination revealed dysarthria, spasticity in both lower and upper limbs, brisk reflexes, especially spastic gait with leg stiffness, and muscle weakness of right hand and both legs, and slight amyotrophy of the distal right hand muscles. Pyramidal tract was involved, and bilateral pectoralis major reflex, palm jaw reflection, Hoffman sign, Babinski sign were also present. Her intellectual function was within normal limits. The MRI of the brain showed a TCC. EMG and nerve conduction velocity recordings showed slightly reduced amplitudes of compound motor action potentials (CMAP) in right common peroneal nerve, and neurogenic change in the upper and lower limbs.

#### Family 2

Family 2: c.4162-10 T > G and c.2163dupT/p. (Ile722fs). These compound heterozygous variants identified in the affected index case were validated by Sanger sequencing; the unaffected father carried the variation c.4162-10 T > G, and the unaffected mother carried the variation c.2163dupT. The point mutation c.4162-10 T > G located at intron 24 has not been reported before and it was predicted to influence the mRNA splicing. The frameshift mutation c.2163dupT was expected to cause early protein truncation, and loss of normal function. This variant in compound heterozygous (c.2163dupT/p.(Ile722fs) and c.7101_7102insT/p.(Lys2368X)) was also reported in a Chinese family [15]. This index case is a 16-year old female. Onset of the disease was at 13 years of age, and the symptoms included stiff gait and intentional tremor of left hand. Subsequently, a spastic cerebellar ataxic gait was developed, and it progressed rapidly. She had severe intellectual disability and did not progress past the junior middle school education. At age 14 years she had two attacks of generalized epileptic seizures, with abnormal EEG at that time. Neurological examination showed increased muscle tone and muscle weakness in lower limbs, brisk reflexes, bilateral Hoffman sign and Babinski sign, cerebellar ataxia, and bilateral pes cavus. MRI showed thinning of corpus callosum and atrophy of cervical spinal cord. EMG showed a prolonged duration of the motor unit action potentials in the right anterior tibialis and the left dorsal interosseous muscle, and slow nerve conductions of motor and sensation were recorded from the tibialis, ulnar, and peroneal nerves.

#### Family 3

Family 3: c.733_734del/p. (Met245fs). Exome sequencing analysis showed that this distinct *SPG11* mutation was in a homozygous form in the affected index case, and the unaffected parents were heterozygous. The c.733_734del mutation was found in both compound heterozygous form and homozygous form in previous studies, suggesting it could be a very ancient mutation or an independent mutational event [3, 8, 15–18]. The p. Met245fs change in exon 4 was predicted to cause early protein truncation. The patient is a 31-year old female with healthy non-consanguineous parents. When she was 20, she showed symptoms of lower limb weakness, accompanied by spasticity which could not be alleviated by treatment with baclofen. At the age of 28, the disease has progressed to a certain stage, and she was not able to walk without support. In addition, she developed slurred speech with frequent choking. Upon examination, she had mild dysarthria, lower limb weakness and spasticity with brisk reflexes and bilateral extensor plantar response, and foot deformity, including pes cavus and hammer toes. Upper extremities were apparently normal. She is cognitively impaired; MoCA scores were 17/30 (impaired in execution, attention, visuospatial ability, and memory). Brain MRI revealed thinning of corpus callosum, especially in the anterior region. EMG of the right tibialis anterior muscle, left medial gastrocnemius, right quadriceps femoris muscle, right biceps brachii muscle, left dorsal interosseous muscle, and left sternocleidomastoid muscle showed chronic denervation characterized by long durations and large amplitudes of motor unit action potentials, with chronic loss of motor units. However, the motor and sensory conduction was normal.

#### Family 4

Family 4: c.3805dupA/p.(Arg1269fs). In the index case, the variants were in a homozygous configuration, but they were in heterozygous form in the unaffected father and mother. The c.3805dupA variation in exon 22 of *SPG11* is a frameshift mutation, which has not been reported. This mutation is predicted to lead to premature transcription termination, and create truncated protein which may lose its normal function. This index patient presented progressive rigidity of the spastic type at the right lower extremity at the age of 13. One year later, the same symptom affected the contralateral left lower limb. As gait difficulties progressed, he developed rapidly deteriorated stiffness in the lower extremities, limb pain and mental impairment. Examination at the age of 16 revealed impaired attention, recall, judgment, and abstract thinking, along with a MMSE score of 23/30. He had a normal muscle bulk, and the power in the lower limbs was normal. Deep tendon reflexes in lower limbs were brisk, with sustained ankle clonus and Barbieski’s sign. Sensory system examination was normal. His gait was spastic with pes arcuatus. Brain MRI revealed a TCC and symmetrical periventricular white matter lesions. EMG showed normal conduction of the sensory action potential, but motor nerve conduction velocity and CMAP amplitude were decreased in the right common peroneal and both tibialis nerves, needle EMG showed a chronic neurogenic pattern in four limbs with denervation signs.

#### Family 5

Family 5: c.317C > A/p. (Ala106Asp) and c.3520 + 3_3520 + 6del. Using Sanger sequencing, these compound heterozygous variants identified in the affected index case were also identified in his affected brother, while the unaffected father carried the variation c.317C > A, and the unaffected mother carried the variation c.3520 + 3_3520 + 6del. The missense mutation c.317C > A located in exon 2 is predicted to lead to an amino acid change at position 106 from Ala to Asp. The deletion mutation c.3520 + 3_3520 + 6del is located in intron 20, and it may affect mRNA splicing. These variants have not been reported before, and Sanger sequencing revealed the correct segregation of this mutation in family members. This index patient was born in a nonconsanguineous family and there had been no complications during gestation or birth. He showed walking difficulties since age 19, and he had prominent tremor in the upper limbs, both resting and during action. He manifested a learning disability requiring special assistance during his compulsory education. Examination at the age of 21 revealed impaired cognitive function. The upper limb muscle had a normal bulk, with an increased muscle tone. Proximal strength was weaker, while hand grasping was normal bilaterally. Severe lower limb spasticity was observed with brisk deep tendon reflexes, but clonus was not observed. The upper limb reflexes were present and normal. Superficial or deep sensation defects were not found. The score of MMSE was 20/30. Brain MRI showed TCC and mild cortical atrophy. EMG studies, including nerve conduction, needle electromyography, and somatosensory and motor evoked potential studies, did not indicate any abnormality.

#### Family 6

Family 6: c.6856C > T/p.(Arg2286X). In the index case, the variants were in a homozygous configuration, but the unaffected mother, father and older sister were shown to be heterozygous. This variation in exon 38 of *SPG11* is a nonsense mutation predicted to lead to a stop codon. Previous study identified the heterozygous form of c.6856C > T variant in a 29 years-old female patient [10]. The index case is a 14-year-old male, with a 2 year history of progressive gait abnormality and bilateral leg weakness. He had a history of cognitive developmental delay and did not speak until age 3 years. The neurological examination showed that he had spasticity in both legs and significant weakness of the bilateral iliopsoas muscles and hamstrings. In addition, he also had slurring of speech, dysarthria, and cognitive decline. His MMSE score was 19/30, and brain MRI showed TCC and moderate cerebral atrophy with slight hyperintensity of the periventricular white matter. Electromyography and nerve conduction studies were normal.

#### Family 7

Family 7: c.7151 + 4_c.7151 + 7delAGTA. In the index case, this variant was identified for being homozygous, but the unaffected father and mother were shown to be heterozygous. Her parents are first cousins. The splicing mutation happened at the splice donor site of intron 39 predicted to affect the splicing of the SPG11 mRNA. This independent homozygous variation has been reported in a Chinese study, and further sequencing results of SPG11 mRNA spanning confirmed that a homozygous alternative donor splice site was generated downstream in intron 39, resulting in a 46 bp insertion and a premature stop codon [15]. The index case is a 19-year-old female; she presented with a 1 year history of progressive gait difficulty and unstable walking, and she felt weakness when climbing the stairs. There were no sensitive disturbances, but she was cognitive impaired, MMSE and MoCA scores were 18/30 and 16/30, respectively (impaired in memory, language and abstract thinking ability). The neurological examination showed weakness and spasticity of the lower extremities with brisk reflexes, bilateral ankle clonus and Babinski sign, and pes cavus deformity. Brain and spinal cord MRI showed a markedly TCC, with some high signal lesions located in the periventricular white matter and bilateral frontal lobe. EMG showed normal motor and sensory conduction, but neurogenic degeneration with loss of motor units in lower limb muscles.

#### Family 8

Family 8: c.3686 + 3_c.3686 + 6delGAGT. In the index case and his affected brother, the variants were in a homozygous configuration, but they were identified in the heterozygous state in the unaffected father, mother, and sister. Her parents are first cousins. This independent homozygous variation is located at intron 21 and leads to a reading frame shift during transcription, resulting in premature termination of the protein product. These variants have not been reported before. The index case is a 17-year-old male; who suffered from progressive weakness and stiffness of the lower extremities for 7 years. He has cognitive impairment; he received the Wechsler Children Intelligence Scale test at age of 14, and the results showed that he had moderate mental retardation. The disease has progressed since the onset and by the time of hospitalization, he was unable to walk independently and was handicapped due to the disturbance in his gait. Neurological examination revealed spastic gait with moderate leg stiffness, and weakness of left quadriceps femoris muscle and tibialis anterior muscle (grade 4/5). Bilateral patellar clonus, positive Babinski’s sign, pes cavus and a scissors gait were also present. Cerebellar function examination revealed impairment. His MMSE score was 20/30. Brain and thoracic MRI revealed thinning of corpus callosum, and remarkable atrophy of thoracic spinal cord. EMG revealed normal motor and sensory nerve conduction velocities, but decreased amplitudes of CMAP, decreased sensory nerve action potentials.

#### Family 9

Family 9: c.1561_1562delAA/p.(Asn521Trpfs). In the index case, the variant was filtered for being homozygous, while his parents carried this variant in the heterozygous state. The parents had normal clinical manifestations, and the patients were not from a consanguineous family. This variation was a novel mutation, which leads to a reading frameshift and results in the truncated at the C-terminus and premature termination of the protein at residue 521. The index case is a 25-year-old male, at 10 years of age he developed a motor disability, and this symptoms progressed to an unstable gait at age 17 years. The clinical signs were characterized by incoordination of lower limbs and a stiff gait at that time, but the symptoms progressed rapidly to a spastic cerebellar ataxic gait. At age 20 years he was wheelchair-bound, accompanied with urinary incontinence. He also had intellectual disability, his school performances were worse than those of his classmates. At age 25 years, he had dysarthric speech and bulbar palsy with dysphagia, and the distal muscles of lower limbs became atrophied and he developed bilateral drop-foot. Due to the dysphagia, his family worried about his feeding problems. Neurological examination showed dementia and dysarthria, spasticity with brisk reflexes in four limbs, and positive Babinski’s sign, cerebellar ataxia, and distal amyotrophy of lower limbs. Brain MRI showed TCC, diffuse white matter abnormalities in the periventricular regions. The EMG showed signs of chronic neurogenic degeneration with large motor units in the lower limb muscles, but the nerve conduction velocity data of motor and sensory nerves and the amplitudes of the action potentials were normal.

## Discussion

We report 9 patients from 2 consanguineous and 7 non-consanguineous marriages showing a combination of progressive spastic gait, accompanied by limb weakness, peripheral neuropathy, or progressive cognitive decline. These cases provide support for the value of NGS as a diagnostic tool in the identification of neurodegenerative disorders, particularly in ARHSP, a disease with high phenotypic and genetic heterogeneity.

The analysis revealed 6 homozygous mutations in the *SPG11* gene, 3 of which were novel and they were predicted to be damaging and cause loss of function of SPATACSIN protein. These mutations were absent in dbSNP147, 1000 Genomes, and EVS. One homozygous mutation (c.733_734del) from family 3 is a frameshift variation, and previous studies showed that this mutation can be existed in both compound heterozygous form and homozygous form, suggesting this is could be a very ancient mutation or an independent mutational event [3, 8, 15–18]. One homozygous mutation (c.6856C > T) from family 6 were nonsence variation, and previous study identified this variant in heterozygous from a 29 years-old female ARHSP patient [10]. We also report here 3 compound heterozygous mutations in the *SPG11* gene. In family 2, the heterozygous frameshift mutation (c.2163dupT) was accompanied by 1 heterozygous missense variation (c.4162-10 T); the former one was previously reported in heterozygous form (c.2163dupT and c.7101_7102insT) in a Chinese family [15]. In the presence of the disease-causing heterozygous frameshift mutation, the role of this missense mutation in heterozygous form cannot be fully interpreted. Although Sanger sequencing showed the correct segregation of this missense mutation in family members and it is predicted to have a great impact on mRNA splicing, its importance in combination with the frameshift mutation is uncertain and remains to be elucidated. In family 5, we found a missense mutation (c.317C > A) combined with a deletion mutation in intron 20 (c.3520 + 3_3520 + 6del). These compound heterozygous mutations were considered to be variants of unknown significance. The results of the genetic testing showed that the same mutation identified in the index case was also found in his brother, this finding could explain his brother’s clinical features. Both the index case and his brother developed the initial symptoms of gait spasticity, which became increasingly severe as the disease progressed. The clinical course in these two affected family members indicates that neurodegeneration is the pathomechanism induced by the compound heterozygous mutations. SIFT and PolyPhen indicate that the c.317C > A variation is deleterious; however, this needs to be further investigated in a larger cohort. In family 1, the compound heterozygous mutations (c.1203delA and c.6284 T > C) were identified; the former mutation is a frameshift variation, and previous studies reported the same mutation in heterozygous in two different cohorts of ARHSP patients [8, 10]; the latter missense mutation was identified in a in Korean cohort with 148 sporadic ALS [14], and this missense variant was found in highly conserved residues and was predicted to be deleterious by in silico analyses. This is a new finding of a mutation in the *SPG11* gene associated with both ARHSP and ARJALS, suggesting the connections of these two diseases at the level of genes. Among the mutations that have been described in *SPG11* gene, frameshift mutations are considered to be the most frequent types [19]. Similarly, our study identified 6 frameshift mutations, accounting for half mutations we found. Furthermore, the newly identified variants in the present study would broaden the spectrum of *SPG 11* mutations in ARHSP patients.

In the present study, we found a wide variety of phenotypic expression in our ARHSP patients, including diversity in clinical presentation. Genetic heterogeneity results in diverse clinical phenotypes. To make the picture more complex, *SPG11* mutations were recently reported to be associated with ARJALS with unusual long disease durations in 2 independent studies [9, 20]. This is another study demonstrating that *SPG11*mutations lead to overlapping clinical phenotypes, with features of both ARHSP and ARJALS. For example, in family 1, the index case first presented with spasticity of right hand, and then she showed symptoms lower limb spasticity and dysarthria; moreover, amyotrophy of the distal right hand muscles were also found. In family 9, after 15 years of disease onset, the patient developed a bulbar palsy in addition to dysarthria, leading to a feeding problem. An evolution to a distal amyotrophy of lower limbs was also identified. Furthermore, in family 1, 2, 3, 4, 7, and 9, the EMG showed neurogenic change with chronic denervation sign, but no fasciculations were seen. All these suggest the involvement of the lower motor neuron and an ALS-like phenotype in a complicated form of ARHSP. The main characteristic of ALS is progressive weakness and signs of dysfunction in both UMN and LMN in one or more body regions. The EMG can find anterior horn signs which are indicative of LMN degeneration and muscle wasting. Although it could be retrospectively argued that a clinical overlap should be considered, there is a clear clinical heterogeneity between the ARHSP and ARJALS. First, the phenotype of these patients is different from the typical phenotype of ALS. This is due to the presence of TCC shown by the brain MRI in all patients, as well as the occurrence of cognitive deficits or mental health problems in some of the pedigrees. Second, in the present study, both LMN and UMN clinical signs were present in same of our patients, but the UMN signs predominated. The presence of TCC and prominent features of upper motoneuron involvement in all of our pedigrees excluded the diagnosis of ARJALS. However, the overlapping phenotypes can blur the line between ARHSP and ARJALS, and we should emphasize the complexity of correct clinical differential diagnosis of diseases linked to *SPG 11* mutations. In addition, previous study performed brain and spinal cord autopsy in patients with complicated HSP carrying *SPG11* mutations and found a neuropathological link between HSP and ALS in terms of neurodegeneration topology; the existence of abnormal accumulations, which is the pathological hallmark of ALS was also found in neurons of SPG11 [21]. Given the common pathological findings in both disorders, including intracytoplasmic granular lysosome-like structures, axonal maintenance and cargo trafficking [22], *SPG11* mutations may contribute to a common pathway in ARHSP and ARJALS. It is possible that ARHSP and ARJALS represent continuing points on the same spectrum, since no clear boundary among the clinical phenotypes is defined in both diseases. Therefore, clinical examination should systematically be accompanied by exome panel testing for mutational screening and electrophysiological investigations, because they are crucial for guiding clinicians to the correct clinical and genetic diagnoses.

## Conclusions

We identified 12 mutations in *SPG11* which are associated with ARHSP in a Chinese population, and 6 variants were novel. Additionally, 1 missense heterozygous variant was found to be carried by both ARHSP patient and ALS patient. To confirm the significance of these mutations in disease pathogenesis, large-scale case-control studies and functional analyses will be required. Clinically and electrophysiologically, some of our ARHSP patients partially shared various features of ARJALS, including combination of both UMN and LMN degeneration. The results contribute to extending of the *SPG11* gene mutation spectrum and emphasizing a putative link between ARHSP and ARJALS.

## Data Availability

The datasets generated during the current study are not publicly available due to hospital policy, which prevents the public dissemination out of concern for patient privacy, but the raw data are available from the corresponding author on reasonable request.

## Abbreviations

ACMG: American College of Medical Genetics and Genomics
ARHSP: Autosomal recessive hereditary spastic paraplegia
ARJALS: Autosomal-recessive juvenile amyotrophic lateral sclerosis
EMG: Electromyography
HSP: Hereditary spastic paraplegia
LMN: Lower motor neuron
MLPA: Multiplex ligation-dependent probe amplification
NGS: Next-generation sequencing
SPG11: Spastic paraplegia type 11
TCC: Thin corpus callosum
UMN: Upper motor neuron
